# Cutaneous Leishmaniasis, Northern Afghanistan

**DOI:** 10.3201/eid1005.030894

**Published:** 2004-05

**Authors:** Richard Reithinger, Khoksar Aadil, Samad Hami, Jan Kolaczinski

**Affiliations:** * London School of Hygiene and Tropical Medicine, London, United Kingdom; † HealthNet International, Peshawar, Pakistan

**Keywords:** Leishmaniasis, *Leishmania tropica*, anthroponotic, control, humanitarian aid, Afghanistan

**To the Editor**: In Afghanistan, most cutaneous leishmaniasis cases are caused by *Leishmania tropica*, which is transmitted anthroponotically by the sandfly *Phlebotomus sergenti* (*1*). Cutaneous leishmaniasis can have devastating effects on local communities because of its clinical symptoms, i.e., large, multiple, or both, disfiguring lesions, that can lead to social ostracism of affected persons (e.g., women are often deemed unsuitable for marriage or to raise children) (*2*). Cutaneous leishmaniasis is considered a low priority disease by international donor agencies because treatment costs are high and the disease does not cause death (*3*).

Data on the effects of cutaneous leishmaniasis in Afghanistan previously have been available only for Kabul city; recent studies have reported an estimated 67,500 cases (*4*). Because of the migration of an estimated 4.5 million infected Afghan refugees returning home from other countries, the sporadic treatment of patients infected with cutaneous leishmaniasis, and limited control of the sandfly vector, *L. tropica* has spread to areas that were previously nonendemic for the disease, e.g., northeastern Afghanistan.

A survey in Faizabad city, Badakhshan Province, was conducted in June 2003 by HealthNet International to collect data on the impact of cutaneous leishmaniasis. Leishmaniasis in this region is transmitted from April to October. The city was divided into 10 districts, and 20 households were surveyed along a randomly chosen transect drawn from the center of each district. A team of experienced medical staff clinically diagnosed cutaneous leishmaniasis (based on the presence or absence of cutaneous leishmaniasis lesions or scars, number of lesions, date of lesion onset) in household members and interviewed them to collect demographic data (gender, age). Because of logistic constraints, parasitologic diagnosis of cutaneous leishmaniasis lesions (i.e., microscopic examination or parasite culture) was not conducted. However, in Afghanistan, skin lesions attributed to causes other than cutaneous leishmaniasis are rare, and experience has shown that clinical diagnosis has a sensitivity and specificity of >80% and >90%, respectively (Reithinger et al., unpub. data). Written approval to conduct the study was obtained from the Ministry of Health. Informed consent was obtained from study participants; all study participants with active cases of the disease were offered free anti-leishmanial treatment at the HealthNet International leishmaniasis clinic.

We surveyed 1,832 people from 200 households; 8.3% (152/1,832) and 7.8% (142/1,832) had active cutaneous leishmaniasis lesions or scars, respectively. Of those persons with cutaneous leishmaniasis lesions, the mean lesion number was 2.4 (range 1–14), the mean lesion size was 2.4 cm (range 1–5.5), and the mean lesion duration (to survey date) was 5.6 months (range 1–11). Active prevalence was not associated with gender (Yates-corrected χ^2^ = 2.16, p = 0.14); 85/152 (56%) of the cutaneous leishmaniasis case-patients were women, and 67/152 (44%) of cutaneous leishmaniasis case-patients were men. Data showed that persons aged ≤15 years were at higher risk of contracting the disease than were persons aged >15 years (odds ratio = 2.23, 95% CI 1.54 to 3.24, Yates-corrected χ^2^ = 19.44, p < 0.001).

Based on population estimates of 65,000 people and observed prevalence, approximately 5,395 cutaneous leishmaniasis case-patients would be found in Faizabad. The low prevalence of scars, compared to the high prevalence of disease, shows that cutaneous leishmaniasis has been introduced into Faizabad only recently (*1*,*4*). Local Ministry of Health records show that the disease was virtually absent (<50 annual cases) in Badakhshan 3 years ago; this information is corroborated by the observation that the mean time since the recovery of surveyed people with cutaneous leishmaniasis scars was 1.5 years (range 0.3–15). Although no attempts were made to identify circulating *Leishmania* sp., the current epidemic is likely caused by *L. tropica* because both men and women are equally affected and younger age groups are at higher risk for cutaneous leishmaniasis than older age groups (*1*,*4*). Current analyses are under way to establish risk factors (e.g., presence or absence of animals, type of house construction) for contracting the disease.

With support from HealthNet International, three leishmaniasis clinics have been established in Faizabad to increase the total number of patients whose illness is diagnosed and treated; to reduce the risk to susceptible persons through the subsidized sale of insecticide-impregnated bed nets; to train and supervise the Ministry of Health staff in diagnosis, treatment, and prevention of the disease; and to implement health education campaigns for patients attending the clinics and the community at large. Hopefully, these activities will prevent the current cutaneous leishmaniasis outbreak from becoming an epidemic, as it has been in Kabul over the past 15 years (*4*,*5*).
